# Supplementary material for: Investigating the ROS Formation and Particle Behavior of Food-Grade Titanium Dioxide (E171) in the TIM-1 Dynamic Gastrointestinal Digestion Model
Source: Nanomaterials (Basel). 2024 Dec 25;15(1):8. doi: 10.3390/nano15010008 (PMC11721885; doi:10.3390/nano15010008)
Supplement: Supplementary file 1 [file nanomaterials-15-00008-s001.zip › nanomaterials-3367971-supplementary.pdf]

Supplementary: Investigating the ROS Formation and Particle Behavior of Food-Grade Titanium Dioxide (E171) in the TIM-1 Dynamic Gastrointestinal Digestion Model

Nicolaj S. Bischoff [1,*], Anna K. Undas [2], Greet van Bemmel [2], Jacco J. Briedé [1], Simone G. van Breda [1], Jessica Verhoeven [3], Sanne Verbruggen [3], Koen Venema [3,†], Dick T. H. M. Sijm [4,5] and Theo M. de Kok [1]

[1] Department of Translational Genomics, GROW Research Institute for Oncology and Reproduction, Maastricht University Medical Centre, 6200 MD Maastricht, The Netherlands

[2] Wageningen Food Safety Research (WFSR), Wageningen University & Research, 6708 WB Wageningen, The Netherlands

[3] Centre for Healthy Eating & Food Innovation, Maastricht University—Campus Venlo, Villafloraweg 1, 5928 SZ Venlo, The Netherlands

[4] Department of Pharmacology and Toxicology, Maastricht University, 6229 ER Maastricht, The Netherlands

[5] Office of Risk Assessment and Research, Netherlands Food and Consumer Product Safety Authority, P.O. Box 43006, 3540 AA Utrecht, The Netherlands

* Correspondence: n.bischoff@maastrichtuniversity.nl

† Current address: Wageningen Food & Biobased Research, Wageningen University & Research, Bornse Weilanden 9, 6708 WG Wageningen, The Netherlands.

Supplementary Materials 1

Gastric Electrolyte Concentrate (GES 10x)
Volume: 5 Liter
Storage: room temperature, 3 months
310g (±3g) Sodium Chloride
110g (±1g) Potassium Chloride
15g (±0,2g) Calcium Chloride Di-Hydrate
4840g (±50g) Demiwater

Small Intestine Electrolyte Concentrate (SIES 25x)
Volume: 10 Liter
Storage: room temperature, 3 months
1250g (±10g) Sodium Chloride
150g (±2g) Potassium Chloride
75g (±1g) Calcium Chloride Di-Hydrate
9400g (±100g) Demiwater
pH 7.0 (±0.5)

Cleaning Solution

Volume: 10 Liter

Storage: room temperature, 3 months

40g (±2g) Sodium Hydroxide

10000g (±100g) Demiwater

200g (±10g) RBS

Trypsin Solution

Volume: 1ml x 100 cups

Storage: -20°C, date of trypsin

200mg (±5) Trypsin

4g SIES 25x

Lipase

Volume: 187mg

Storage: 4°C, date of Lipase

187mg per cup = 5625units

Fed run = 5625 units

Fasted run = 1125 units

Pepsine

Volume: 36mg

Storage: -20°C, date of pepsine

36mg per cup = 90.000 units

Fed run = 90.000 units

Fasted run = 18.000 units

Pancreatin

17,5g pancreatin to 250ml water

All from Sigma Aldrich/Merck group, except RBS: Carl Roth

Bile

15g bile to 375ml water (Sigma)

Diluted GES

Volume: 1 Liter

Storage: room temperature, 3 months

102g (±5g) GES 10x

898g (±1g) Demiwater

Diluted SIES

Volume: 2 Liter

Storage: room temperature, 3 months

87g (±2g) SIES 25x

1913g (±10g) Demiwater

Sodium Bicarbonate 1M

Volume: 2 Liter

Storage: room temperature, 3 months

168g (±2g) Sodium Bicarbonate

1940g (±20g) Demiwater

Hydrochloric Acid 1M

Volume: 1 Liter

Storage: room temperature, 3 months

250g HCl 4M

750g Demiwater

Sodium Hydroxide 1M

Volume: 1 Liter

Storage: room temperature, 3 months

40g (±0,4g) Sodium Hydroxide

960g Demiwater

Sodium Citrate buffer 0.1M

Volume: 1 Liter

Storage: room temperature, 3 months

29,4g (±0,5g) Tri Sodium Citrate Dihydrate

970,6g Demiwater

pH 7.0 (±0.2)

Sodium Acetate buffer 1M

Volume: 1 Liter

Storage: room temperature, 3 months

87.1g (±1g) Sodium Acetate tri hydrate

900g Demiwater

21.6g (±0,5g) Acetic Acid (toevoegen in zuurkast)

pH 5.0

All Sigma Aldrich/Merck group, except hydrochloric acid: VWR international

Supplementary data

Supplementary Table S1: spICP-MS specification for the characterization of E171.

Instrument Parameter	***Operating Conditions***
Power	1600 w
Nebulizer	Quartz concentric PFA-st3 1 ml/min Ar (back pressure 394 kPa Perkin Elmer N 8152378 REV A)
Spray chamber	Cyclonic high purity spray chamber (Perkin Elmer N8152424 REv A)
Flow nebulizer	0.31 ml/min
Peristaltic pump	-60 rpm
Mode	Standard (Nano application)
QID	-14V
Selected masses	48 Ti
Dwell time	100 us
Sampling time	60 sec
TE	9.07
Density	3.9 (g/cm^3)
Mass fraction	60%

Supplementary Table S2: DLS results from E171-aq measurements before and along the TIM-1 GI digestion

		Mean HD (nm)			***Average Intensity (%)***				
Sample	**pH**	**Peak 1**	**Peak 2**	**Peak 3**	**Peak 1**	**Peak 2**	**Peak 3**	**PDI**	**Zeta (mV)**
E171-aq	7.2	353 ± 5	4994		99.1	1.9		0.19	-31.4
Meal	4.5	701 ±26	-	-	100	-	-	0.89	-26.3
IE1	8.2	542 ±24	5541 ±27	-	99.3	0.8	-	0.52	-29.1
IE2	8.2	478 ± 52	5560	-	99.6	0.8	-	0.54	-50.4
IE3	8.2	618 ±101	5317 ±173	150	94.4	3.6	4.0	0.42	-52.2
IE4	8.0	720 ± 39	5273 ± 165	121 ±91	88.6	5.2	6.3	0.54	-48.3
IE5	7.8	750 ± 11	4746 ±670	92 ± 15	85.6	7.8	6.6	0.67	-41.8
IE6	7.8	758 ±11	4992 ±100	95 ± 37	84.7	7.6	7.8	0.62	-34.7

Supplementary Table S3: DLS results from E171-yog measurements before and along the TIM-1 GI digestion

		**MEAN HD (NM)**			**AVERAGE INTENSITY (%)**				
**SAMPLE**	**pH**	**Peak 1**	**Peak 2**	**Peak 3**	**Peak 1**	**Peak 2**	**Peak 3**	**PDI**	**Zeta (mV)**
**E171-YOG**	5.9	6115 ±1017	-	-	100	-	-	0.32	1.55
**MEAL**	6.2	4893 ± 943	-	-	100	-	-	0.68	1.68
**IE1**	7.4	1400 ± 114	5391 ± 49		93.6	6.5	-	0.45	-25.55
**IE2**	7.5	1565 ± 214	4922 ± 806	254.6	80.5	16.6	3.6	0.46	-27.16
**IE3**	7.8	1233 ±105	5028	-	91.4	7.3		0.54	-24.65
**IE4**	7.8	1436 ± 129	4623 ± 39	467	83.6	13.7	3.3	0.48	-25.86
**IE5**	7.7	1603 ±121	4460 ± 35	-	66.4	33.7	-	0.43	-25.10
**IE6**	7.8	1911 ± 359	4620 ± 140	488	73.5	24.7	3.5	0.38	-23.71

Supplementary Table S4: Summary of spICP-MS of TIM-1 dE171-aq and dE171-yog samples

**DE171 -AQ**						**DE171 -YOG**					
	**Mean (nm)**	**D10 (nm)**	**D50 (nm)**	**D99.5 (nm)**	**Particles < 100 nm (%)**		**Mean (nm)**	**D10 (nm)**	**D50 (nm)**	**D99.5 (nm)**	**Particles < 100 nm (%)**
**MEAL**	367	210	355	724	1	**Meal**	377	198	337	668	1
**IE1**	136	105	321	513	54	**IE1**	286	137	269	566	5
**IE2**	344	192	338	679	2	**IE2**	294	143	273	588	4
**IE3**	322	131	300	655	7	**IE3**	313	140	269	581	4
**IE4**	326	170	319	688	2	**IE4**	338	164	301	640	3
**IE5**	314	163	322	683	3	**IE5**	328	157	292	616	3
**IE6**	323	174	334	728	2	**IE6**	289	143	270	580	4

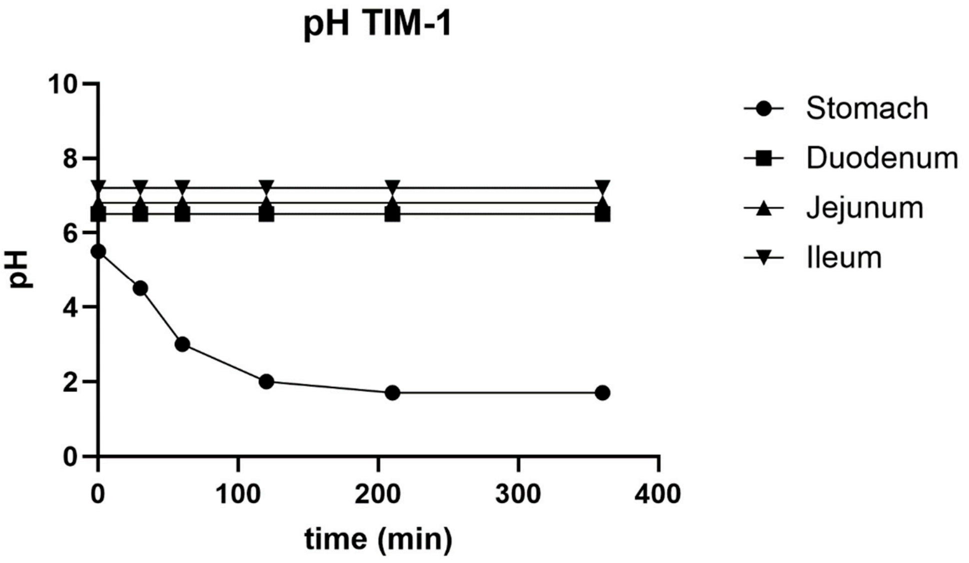

Supplementary Figure S1: Display of pH levels of the TIM-1 model over time and different compartments, including the stomach, duodenum, jejunum and ileum

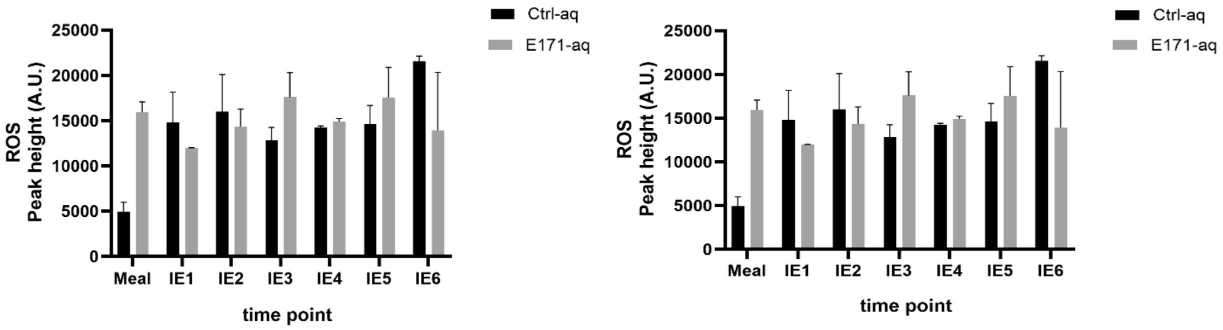

Supplementary Figure S2: ESR measure to quantify ROS signal for E171-aq and E171-yog and their respective controls when stimulated with 1 mM H_2O_2. The addition of 1 mM H_2O_2 increased the ROS signal in both control and E171 exposure group for E171-aq and E171-yog, indicating that the addition of 1 mM H_2O_2 does not increase the capacity of E171 to form ROS following in vitro digestion in the TIM-1 model.

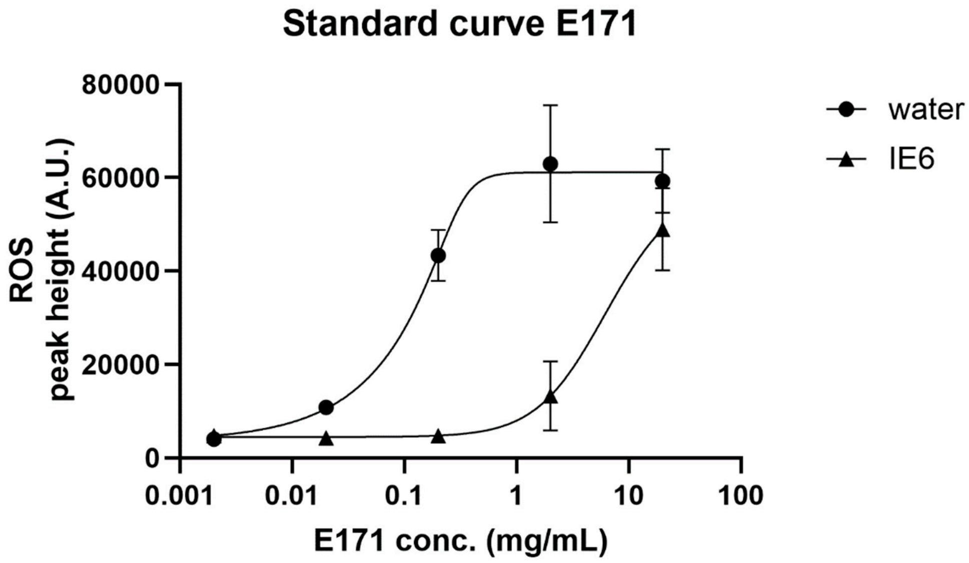

Supplementary Figure S3: E171 standard curve in aqueous dispersion and ileal efflux dispersion, with various concentration of E171. E171 can induce ROS in aqueous solution at lower levels than in the ileal efflux.
